# Bridging the gap: Understanding the needs of community health volunteers to enhance palliative care delivery

**DOI:** 10.1177/26323524261473935

**Published:** 2026-08-23

**Authors:** Mark Donald Mwesiga, Lisa Christine Irumba, Christine Siew Lan Low, Christie van Veen, Nena van de Weijer, Suzanne Gros, Dorien van de Ven, Wout Linsen, Joyce Zalwango, Dianah Basirika, Rose Kiwanuka, John Muwonge, Mwazi Batuli, Gloria Kirungi Kasozi

**Affiliations:** 1 Palliative Care Association of Uganda, Kampala, Uganda; 2 Centre for Rural Health, 117152 University of Tasmania School of Health Sciences, Launceston, TAS, Australia; 3 School of Health Studies, 6032 HAN University of Applied Sciences, Arhem, Gelderland, Netherlands; 4 127812 International Programs, Hospice Africa Uganda, Kampala, Central Region, Uganda; 5 Lweza Community Health Programme, Kampala, Uganda; 6 108122 Islamic University in Uganda, Mbale, Eastern Region, Uganda; 7 183054 Nkumba University, Entebbe, Central Region, Uganda

**Keywords:** palliative, need, community health volunteers, volunteer, CHV, VHT

## Abstract

**Background:**

Community Health Volunteers (CHVs) serve as lay providers who deliver essential health services and bridge the gap between underserved populations and formal healthcare systems. In Uganda, these volunteers act as the primary point of contact and a vital intermediary between rural communities and health facilities. They facilitate community-based care, manage referrals, and provide health education, offering a cost-effective strategy to mitigate human resource shortages in low-income health systems. Although CHVs constitute the backbone of primary healthcare, systemic barriers such as poor integration, high attrition, and insufficient training frequently undermine their effectiveness. These challenges particularly stifle service delivery in rural districts where formal infrastructure is limited.

**Objective:**

This study aimed to explore the specific needs of CHVs to inform targeted interventions that strengthen capacity building and improve palliative care outcomes in community settings.

**Design & Methods:**

This qualitative study employed semi-structured interviews with eight CHVs from Mutungo Parish, Wakiso District, selected through purposive sampling. The interview transcripts underwent rigorous thematic analysis following Bingham and Witkowsky (2021) structured five-step analytical framework, with data management and coding procedures implemented in Microsoft Excel to maintain methodological consistency and analytical transparency.

**Results:**

Four key themes were identified through thematic analysis. First, regarding training and education, CHVs reported insufficient palliative care training and specifically requested competency-based refresher courses. Second, concerning resources, participants identified critical shortages in tangible, operational resources which includes medical supplies, transportation funds and material support in providing care. Third, in communication and coordination, there was a demonstrated need for smartphones with data plans to facilitate care coordination. Finally, on the workforce sustainability, analysis revealed excessive patient loads stemming from workforce shortages that compromised service delivery.

**Conclusion:**

The findings reveal urgent, context-specific gaps in Uganda’s community-based palliative care system. Addressing these through tiered training, resource provision, and workforce expansion could significantly improve care quality.

## Plain language summary

Community Health Volunteers (CHVs) are local volunteers who provide essential health services and act as a bridge between their communities and the formal healthcare system. In Uganda, they play a particularly important role in palliative care, supporting seriously ill patients with pain management, emotional support, and end-of-life care, especially in rural areas where healthcare facilities are limited. This study explored what CHVs in Wakiso District, Uganda, need to do their jobs more effectively. Researchers conducted in-depth interviews with eight CHVs to understand the specific challenges they face in delivering palliative care. Four key challenges were identified. First, most CHVs received only basic health training that did not adequately prepare them for the complex demands of palliative care. They expressed a clear need for more structured, ongoing training in areas such as pain management and psychosocial support. Second, CHVs consistently reported shortages of basic supplies, including medical equipment, protective gear, and transportation funds. Without these, reaching patients in remote areas and providing safe, effective care becomes very difficult. Third, CHVs relied heavily on informal communication methods such as phone calls and motorbike transport to coordinate patient referrals, with no structured system in place. Access to smartphones and data plans would significantly improve how they share information and coordinate care. Fourth, many CHVs were overwhelmed by large patient caseloads due to a shortage of volunteers. This, combined with inadequate and often delayed pay, led to burnout and threatened the sustainability of the volunteer workforce. Addressing these gaps through better training, resource provision, improved communication tools, and fairer compensation could meaningfully strengthen palliative care delivery in Ugandan communities.

## Key statements

### What is already known about the topic?

• Community Health Volunteers (CHVs) are essential for extending palliative care into community and home settings, especially in low-resource countries like Uganda.
• International health bodies widely recommend designating CHVs specifically for palliative care to improve access to dignified end-of-life care.
• While a range of CHVs role exist in the community, the evidence bases for their effectiveness, in terms of patient and service use outcomes, is not known.

### What this paper adds

• Provides rich, contextualised qualitative evidence on the lived experiences and unmet needs of community health volunteers delivering palliative care in Uganda.
• Identifies specific support needs of CHVs in four major areas: training and education, resources, communication and coordination, and workforce sustainability.
• Offers practical, evidence-based recommendations such as revising training standards, harmonizing efforts among partners, creating a national CHV database, and introducing performance-based financing to enhance sustainability and effectiveness of CHV programmes.

### Implications for practice, theory or policy

• Future CHV programme development and evaluation should include patients, caregivers, and other stakeholders to ensure CHV roles are shaped by community needs and preferences.
• Standardised, competency-based palliative care training integrated into national curricula, with ongoing refresher courses and mentorship.
• Policy development should address current limited evidence, prioritize CHVs support needs, and build stronger institutional systems for referral, supervision, and digital inclusion.

## Introduction

Community Health Volunteers (CHVs) are defined as lay individuals who deliver health services voluntarily within a structured framework, without formal contractual obligations or financial remuneration.^1,2^ Typically drawn from the communities they serve, CHVs play an important role in bridging gaps between local populations, caregivers, and formal healthcare systems, particularly in underserved areas with limited access to health services.^1^ Since the Alma Ata Declaration in 1978, CHVs have been formally recognised as the third workforce within the ‘Human Resources for Health’ framework.^3^ Consequently, numerous countries, particularly in Asia and Africa, have integrated CHVs into their public health strategies to advance healthcare objectives.^2,4^ CHVs primary responsibilities include health promotion, disease prevention, and the provision of culturally appropriate care.^5–7^ Their role is critical in bridging the gap between health facilities and underserved populations, particularly in low-resource settings where human resource constraints and limited infrastructure hinder access to care.

In Uganda, CHVs are integral to the Village Health Team (VHT) initiative, a key component of the national healthcare system, especially in rural and hard-to-reach regions. Established by the Ministry of Health (MOH) of Uganda in 2001,^5^ the Village Health Team programme was embedded within Uganda’s National Health Strategic Plan (2001–2006), reflecting the government’s commitment to a client- and community-centred approach.^8^ Selected by their communities, these volunteers serve as intermediaries between residents and formal healthcare providers.^9^ CHVs serve as the frontline of primary healthcare, conducting household visits, delivering health education, and facilitating referrals to health centres.^9,10^ Their responsibilities extend to maternal and child health, immunization promotion, malaria prevention, and management of common illnesses. Additionally, they play a vital role in palliative care, offering basic pain management, psychosocial support, and end-of-life care for patients with chronic and life-limiting conditions, services that are often unavailable in remote areas.

However, despite their critical role, CHVs encounter persistent challenges that hinder their effectiveness. These include high attrition rates, with studies reporting 30-60% dropout rates within the first two years of service, primarily due to lack of financial incentives, inadequate medical supplies, and limited training opportunities.^10–15^ Reports from the Uganda Ministry of Health further highlight systemic weaknesses in training programmes, supervision mechanisms, and motivational strategies for CHVs.^16,17^ Compounding these challenges is the near-total exclusion of palliative care from in CHVs’ training. Less than 10% of CHVs receive instruction in pain management, despite Uganda’s high burden of chronic illnesses.^14,18^ While the government has implemented measures to strengthen the CHVs programme, focusing on capacity-building and volunteer welfare, gaps remain in addressing the specific demands placed on CHVs working in specialised fields such as palliative care.^14^

Thus, this study aimed to identify the specific needs of CHVs delivering palliative care in community settings, with the goal of informing targeted interventions to strengthen capacity building and improve patient care outcomes.

## Methods

This study utilised a qualitative, descriptive approach to understand the needs of CHVs to improve palliative care service delivery within Ugandan communities.

i. Selection and Description of Participants

A purposive sampling method was used to recruit CHVs. The principle of “meaning saturation” proposed by Hennink and colleagues (2016) was employed to guide sample size.^19^ The inclusion criteria ensured participants were active CHVs known by the Local Council (indicating government registration), had at least one year of experience, and were actively involved in palliative care (working with patients weekly). Conversely, CHVs with less than a year of experience or who did not speak sufficient English for in-depth interviews were excluded. This decision aimed to include volunteers with a strong understanding of their role and fluent English for clear communication, avoiding potential meaning shifts through translation.

ii. Data collection

Semi-structured, face-to-face interviews were conducted with CHVs from 30^th^ October 2023 till 1^st^ December 2023 to gather in-depth information about their experiences and needs in delivering palliative care service. The interview guide was developed following a comprehensive literature review, through which key thematic areas were identified to guide data collection: training, education, resources, communication, coordination, and individual factors. The guide was subsequently refined through collaborative consultations with a multidisciplinary team experienced in qualitative methodology and an experienced CHV, to ensure methodological rigour and cultural appropriateness. The final interview guide comprised four sections of open-ended questions corresponding to these key areas and was reviewed and approved by the Hospice Africa Uganda Research and Ethics Committee (HAUREC) prior to data collection. The interviews were audio-recorded and transcribed verbatim. During the interviews, in-interview verification was employed, whereby the interviewer paraphrased participants’ responses to verify understanding and allow for clarification. Participants’ anonymity was maintained by assigning unique identifiers to each participant and deleting audio recordings upon completion of transcription. Three authors were involved in data collection: NW, CV, and/or SG led the interviews, while another author observed, took notes, and managed the recording. Each interview was scheduled for 60 minutes but lasted between 45 and 90 minutes. Participants were compensated with 30,000 Ugandan shillings for their time.

iii. Data analysis

This study used an inductive thematic approach which allow participants lived experiences to shape the meaning and content of the findings. Consistent with Richards and Morse's (2013) principle of methodological congruence, the analytic process remained grounded in the data to ensure that the interpretive outcomes reflected participants’ perspectives rather than the imposition of pre-existing theoretical constructs.^20^ The analytical process followed the structured five-phase iterative cycle of qualitative analysis as developed by Bingham and Witkowsky (2021)^21^ and further elaborated by Bingham (2023).^22^ The interview transcripts were independently analysed and coded by three authors (NW, CV and SG), with findings discussed collaboratively with the broader research team, including LI, DV and WL. The analysis began with: (1) familiarization, involving repeated reading and deep immersion in the data to gain a holistic understanding of participants' lived experiences. Using Microsoft Excel (Version 16.0), key words, phrases, and concepts relevant to the research question were identified within the transcribed texts. This was followed by (2) initial coding, whereby the data were systematically segmented into meaningful units and open codes were assigned to each unit, driven entirely by the content of the data rather than pre-existing categories. Codes were generated inductively, remaining close to the participants’ own language and meanings. The process then moved to (3) searching for themes, where the generated codes were reviewed, grouped, and refined to identify coherent patterns across the dataset, allowing thematic categories to emerge organically from the data. In (4) reviewing and defining themes, the emerging themes were iteratively reviewed against the coded data extracts and the full dataset to ensure they accurately represented the participants’ experiences. Themes were then named and defined to capture their essence and relevance to the research question that enables a deeper and contextually grounded interpretation of the data. Finally, (5) producing the analytical narrative involved synthesizing the findings into a coherent account, supported by illustrative participant quotations and contextual interpretations which culminating in a comprehensive narrative that directly addressed the research question. Throughout this process, investigator triangulation was employed, whereby independent coding by three team members (NW, CV and SG) was followed by collaborative discussion and consensus-building with the broader research team. This iterative process of discussion and reflection enhanced the credibility and trustworthiness of the analytical outcomes. Member-checking was conducted whereby participants were offered the opportunity to review and verify their interview transcripts; however, logistical constraints inherent to the field setting limited full participation in this process.

## Results

### Social demographics of respondents

Table.1 provides a demographic overview of the eight CHVs who participated in the qualitative research. The respondents were roughly distributed across three villages (Lweza A, Lweza B, and Kigo) within Mutungo parish, Wakiso district, Uganda. In terms of gender, the majority (75%) were female, while 25% were male. The age range of the participants was diverse, covering from 26 to 61 years old. Regarding work experience, the CHVs demonstrated a range of experience levels, with approximately 37.5% having 1-5 years of experience, 37.5% having 6-10 years of experience, and 25% having more than 10 years of experience.

**Table 1.** Demographic characteristics.

Category	Subcategory	n (%)
Gender	Male	2 (25.0%)
	Female	6 (75.0%)
Village	Lweza B	3 (37.5%)
	Lweza A	2 (25.0%)
	Kigo	3 (37.5%)
Work experience (years)	1-5	3 (37.5%)
	6-10	3 (37.5%)
	>10	2 (25.0%)

## Themes

The following results document the lived experiences and inductively identified unmet needs of CHVs in delivering palliative care within their communities. The findings of this study highlight several key challenges faced by CHVs in delivering palliative care, as well as their expressed needs to improve service delivery. Four major themes were constructed from the data: 1) Training and Education, 2) Resources, 3) Communication and Coordination, and 4) Workforce Sustainability. Each theme reflects systemic gaps that, if addressed, could significantly enhance the capacity of CHVs to provide effective palliative care.

### Training and education

The majority of the interviewed CHVs reported receiving basic training that covered essential child and adult health interventions, including managing malaria and pneumonia cases, administering medications, and referring patients to health centres. Their training typically included three key components^1^: under-five child health (U5MR), which includes recognizing malaria and pneumonia symptoms and providing appropriate antimalarials^2^; diagnostic skills such as conducting malaria tests and proper sample collection; and^3^ data reporting procedures for accurately recording patient information to be shared with health facilities. However, while CHVs received introductory instruction in communication and counselling, many described this training as inadequate for addressing real-world caregiving challenges, particularly in palliative care. Volunteers consistently identified critical gaps in their skills and knowledge which underscore the urgent need for enhanced training in symptom management, psychosocial support, and chronic disease care within palliative care.

“I got training on how to consult and recognise people (with palliative care needs) in the community…We are missing the skills (referring to palliative care knowledge & skills) because some of us (referring to the people in the community) are not in good condition. I think we could get some skills that help us, so that when we have skills, we can teach others because volunteers want to help others because it is in our blood.” (Participant R4)

“Yeah, we want to get more training (referring to palliative care knowledge & skills), beyond what we have.” (Participant R8)

Although respondents acknowledged the need for further training, many found it challenging to articulate precise gaps in their current skill set. However, several participants pinpointed critical areas for skill development, particularly in providing psychosocial support for patients with chronic illnesses and delivering practical life skills training to better assist both patients and their families. CHVs frequently observed that chronic illnesses create complex challenges that extend far beyond medical needs, in which patients and families often require comprehensive support to manage both the practical realities of daily living and the profound psychosocial impacts of long-term illness.

“So, it would be better when we learn other skills (referring to life functioning skills), so we come and equip them, and they be able to run their life.” (Participant R1)

“…some patient may come as HIV patient and they want something, they want more people to understand them because someone can come when stressed, you forget that kind of diseases, you can feel if you are going to die at that moment. I need more education in that.” (Participant R7)

Participants described considerable variation in the structure and frequency of palliative care training they had received. Two distinct patterns were identified: some participants described receiving a condensed model of training delivered over a single day each month, focused on basic palliative care identification and counselling skills, while others reported an initial intensive week-long training supplemented by quarterly refresher courses. These accounts point to meaningful differences in training duration, frequency, and content depth across participants’ experience.

“One day a month training (referring to palliative care identification and counselling skills)” (Participant R4)

“The training I did it took like a week, a whole week but many times, we have training in here. they can call us, and we go back for the training and the training just to refresh us…the refreshment course is like four times a year.” (Participant R5)

Participants also described a perceived decline in palliative care training opportunities over time, which they attributed to funding constraints. Several noted that follow-up or refresher training had previously been available but had since stopped or become less frequent. Participants linked this reduction to gaps in their current skills and to difficulties they faced in providing adequate care to patients.

‘‘These trainings were there but now we are not having because of funds. We used to have trainings but now we do not have.’’ (Participant R2)

### Resources

CHVs consistently identified critical shortages in the tangible resources needed to carry out their roles, spanning transportation, medical supplies, protective equipment and basic material support. Transportation was identified as a major barrier, with all CHVs identifying mobility constraints as a key barrier to reaching patients in remote communities. The absence of reliable and affordable transport options forced many volunteers to depend on physically demanding methods like walking long distances or expensive alternatives such as boda-boda (motorcycle taxi) services, which were often unaffordable.

“Then there is a challenge when sometimes you have to get boda-boda and there is no money, that challenge is there.” (Participant R2)

“I have few of them [referring to options for getting patients medical care at home], because it means money to call a doctor, to come to the home, …Like all of them, they need us to get their own doctor. It's good but it's very expensive here to be, to having a doctor. If you have, if you don't feel well, to call the doctor to come to your home. It's very expensive, in our country, it's not easy. At least they have to meet them when they going to get the medicine from the hospital.” (Participant R3)

The CHVs proposed two potential solutions. These include direct transport allowances or provision of motorcycles to improve accessibility. This transportation support would enable them to travel independently to reach patients in need, especially those who are living in rural and remote areas.

“I use the boda-boda, yeah, because I don't have money for uber. I don't have a car. I just get a boda-boda and then I go to that person. They are very far. If I want to take her into the hospital, I have to use the boda-boda.” (Participant R3)

“But for us it would be good if they gave us motorcycle because even bicycle, it is not good. You cannot ride on this. the roads are not nice. if you have a motorcycle you can move on…if you have a motorcycle you can move on.” (Participant R5)

A significant proportion of CHVs reported facing cultural expectations to provide material support beyond healthcare services, including in-kind assistance such as food or financial help. Many described how community members often assume volunteers have the capacity to offer this type of aid, creating substantial pressure on CHVs who frequently lack the resources themselves. This disconnect between community expectations and CHVs’ actual capacity highlights a critical challenge in their role.

“We also need money because the people will come at your place and think we have money, what we are going to give them for help, but we don't have…need like other care, you know, like the ones we use at home, like some soap and food.” (Participant R5)

In addition to transportation and material supports, several essential items were identified to support CHVs in effectively navigating challenging rural environments. Approximately half of the respondents expressed a need for essential supplies and equipment, such as rain gear, flashlights, umbrellas, rain jackets, or boots. These essentials are necessary for navigating difficult terrain, particularly in adverse weather or at night, particularly in remote areas with limited infrastructure. As exemplified by some participants, equipping CHVs with appropriate tools and supplies can significantly enhance their ability to provide timely and effective care in challenging circumstances.

“When it is raining, we don’t have umbrella, raincoat, boots because we are walking in mud which slows me down.” (Participant R6)

“Beside rain, one thing we have, some lights, so that at night, we go there, and the power is off, you are walking in the dark and cannot see.” (Participant R5)

Furthermore, medical supplies, including first aid kits and gloves, were another critical need, with their absence hindering effective care. A first aid kit, equipped with basic medications, including analgesics for pain relief, was considered essential for providing initial symptom management and alleviating suffering in palliative care patients before referral to higher-level healthcare facilities. Similarly, basic protective supplies such as gloves were deemed indispensable for delivering safe hands-on care to palliative care patients, many of whom present with complex illnesses and open wounds that require careful handling to prevent cross-infection. The consistent absence of these fundamental supplies not only compromises the quality of palliative care that CHVs are able to provide but also exposes both patients and caregivers to preventable risks. Hence, this can undermine the broader goals of community-based palliative care delivery.

“Me, I don’t give them first treatment. That is something missing, I don’t know where we should get but it would serve the purpose, if we would have some aid. First aid to give them, those who have other complications we refer them.” (Participant R2)

“The challenge that I mostly experience, drugs, cause a patients can come to you in pain. What am I going to do? I don't have any single drug. That is a challenge I am getting.” (Participant R7)

### Communication and coordination

The participants highlight both the strengths and limitations of communication and coordination in CHV networks. While CHVs actively engage in patient assessments and referrals, their ability to escalate complex cases depends heavily on informal, ad-hoc communication channels. This is done primarily through phone calls and motorbike (boda-boda) transport, rather than a structured, reliable system. This fragmented approach risks inefficiencies, as referrals may be delayed or lost without standardised protocols.

“We go to see the patient, we get information from them, and when we cannot handle, we send them to a higher level…we do communicate on phone and sometimes we go there when the hospital is a little bit near…we communicate with other volunteers through the phones and boda-boda riding.” (Participant R8)

The participants also stressed the need for digital communication tools, such as smartphones or laptops, to strengthen coordination, collaboration, and real-time information sharing within the healthcare system. Many specifically stressed the importance of having smartphones equipped with sufficient call time and internet connectivity to perform their roles efficiently. These devices would enable CHVs to maintain real-time communication with other healthcare providers, facilitating seamless referrals, consultations, and updates on patient care.

“Me, I cannot afford to buy a smartphone. Sometimes when you reach out this side, you want to photograph, then you send it to your boss. This is not good phone [shows her phone]. You end up failing to send the information very fast.” (Participant R2)

While most discussions focused on smartphones, several CHVs also recognized the importance of laptops as essential tools for effective documentation and data management. Many emphasized how digital tools could significantly improve their work by streamlining processes and safeguarding critical health information. As for instance, maintaining accurate health records, tracking patient care trajectories, and ensuring seamless information sharing across different levels of the healthcare system.

“I know that there are some good apps of which you can use or install on our phones and that would keep the data and keep sharing to different people like the doctors and different levels of care…They’re not providing like a laptop. So, you find that you write on a piece of paper of which at any time you can lose it.” (Participant R1)

### Workforce sustainability

A significant proportion of CHVs reported feeling overwhelmed by their patient caseloads, with many communities facing a critical shortage of volunteers to meet healthcare demands. The combination of limited CHV numbers and large patient populations created substantial challenges in delivering comprehensive care to all individuals in need. Many of them described struggling to manage their workload effectively, particularly when addressing palliative care cases involving patients experiencing acute pain, advanced disease symptoms, or those requiring urgent referrals to hospice or specialist palliative care services. The burden was further compounded by the emotionally demanding nature of palliative care work, including accompanying patients and families through end-of-life processes with limited professional support.

“Yes, there are too many people to give care. You find that I am alone in that area of which there are even more than 10,000 homes. So, end up not capturing all the data for some people. So, we need more people. So, I am being alone, that is a challenge… Sometimes or everyday, some are in pain, sometimes you refer, or time taken up by another person, so sometimes you don’t have enough volunteers to run the whole village, that way, we cannot give good care.’’ (Participant R1)

Compensation was another significant concern, with many CHVs struggling to sustain themselves due to inadequate or delayed payments. Many participants expressed difficulty in remaining motivated without receiving adequate compensation. Some reported insufficient salaries to support themselves and their families, while others indicated that promised salaries were either not paid or paid too late. A few participants even received no salary at all. These financial challenges highlighted the significant need for fair and timely compensation to support CHVs in their roles. For example, participant describes receiving minimal compensation, often in the form of gifts, which is insufficient for their basic needs. Likewise, another participant emphasises the importance of financial support in maintaining motivation.

“I have been volunteering, living from a gift at the end of the week, or at the end of the month but whatever they are giving, it is not quite enough to sustain yourself. So, it’s a challenge on my side.” (Participant R1)

“We get payment when we submit it (the patient’s report). They give us some money, but it doesn’t come direct, like you submit it in November, and you will get it next year. It delays. It doesn’t help much…but yeah, you give care with love, but it is sometimes hard to be motivated if you didn’t get any money or something.” (Participant R8)

## Discussion

This study explored the challenges faced by CHVs in delivering palliative care, identifying four key themes: Training and Education, Resources, Communication and Coordination, and Workforce Sustainability. These findings highlighted the distinctive systemic barriers that hinder CHVs from performing their roles in palliative care effectively.

### Training gaps and the need for standardised capacity building

The study reveals a critical disconnect between CHVs’ foundational training and the complex realities of palliative care delivery. Participants’ accounts point to an uncoordinated approach to capacity building, where training duration, frequency, and content depth appear dependent on local implementation rather than standardised protocols. While CHVs receive competency-based instruction in malaria/pneumonia management and data reporting (components aligned with Uganda’s iCCM framework), their preparation for palliative-specific challenges remains inadequate. This mirrors regional findings from Kenya^23^ and Uganda’s own CHV’s assessments,^24^ where basic training fails to address symptom management, psychosocial support, and chronic disease care. This aligns with broader scoping reviews on CHWs in palliative care, which found variability in training content and insufficient standardisation.^25^ To bridge these training gaps, Uganda must transition to a standardised, competency-based training system tailored to palliative care realities. The BIRCH Project provides a foundation by establishing unified national guidelines and clarifying the complementary roles of CHVs.^26^ Training should be standardised and extended (10–14 days plus refreshers), supplemented by digital learning tools and district-level mentorship. Institutional systems also require clear national protocols for referral and supervision, mHealth tools for task tracking, and structured monthly supervision with health facilities.^26–28^

### Resource scarcity leading to multidimensional barrier

Transportation was identified as the most acute resource challenge, with CHVs incurring out-of-pocket costs for boda-bodas, a finding consistent with studies in rural Uganda.^29^ The lack of rain gear and flashlights further compounds accessibility issues during inclement weather or nighttime visits, echoing Musoke’s (2019) observation that such logistical hurdles reduce service coverage by up to 40%.^10^ The lack of financial and logistical support significantly reduced their capacity to perform essential home visits and timely referrals. Mwaniki and colleagues (2025) highlighted that inadequate transport, coupled with limited sustenance and protective gear, led to fatigue and decreased coverage in remote areas, reinforcing transportation as a systemic barrier rather than an individual inconvenience.^30^​

### Communication and data management

The study reveals both resilience and fragility in CHV communication systems. While volunteers adapt through informal coordination, reliance on ad-hoc methods exposes systemic vulnerabilities. This aligns with evidence showing that digital health tools can mitigate such gaps by enabling real-time documentation and coordination.^31^ The absence of standardised referral protocols jeopardises care continuity, delaying interventions and risking patient outcomes.^32^ CHVs expressed an urgent need for digital tools such as smartphones with airtime and internet access to support real-time communication and data sharing. This mirrors findings from Ethiopia, where paper-based reporting causes delays and data loss.^33^ However, the practical feasibility of deploying such tools must be carefully considered within Uganda’s infrastructural realities. The Uganda National Household Survey reports that only 18.9% of households are connected to the national electricity grid, which stress the need to prioritise solar-compatible, low-power digital devices alongside smartphones to ensure that technology-based solutions are genuinely accessible to CHVs across rural and peri-urban settings.^34^ The persistent digital divide nonetheless risks excluding some volunteers from decision-making loops. Ensuring digital inclusion through provision of appropriate devices, connectivity, training, and institutional support must therefore be prioritised as a key component of health system strengthening.^35^

### Workforce shortages, burnout crisis, and precarious finances

The findings reveal serious workforce shortages among CHVs, with many volunteers reporting overwhelming caseloads that compromise their ability to deliver quality care. This resonates with the National Village Health Team Assessment in Uganda (2015), where excessive workloads directly undermine service delivery.^14^ Without addressing these capacity constraints, communities risk continued gaps in essential health services, particularly for vulnerable patients with urgent palliative care needs. The problem is global. Jaskiewicz and Tulenko (2012) demonstrated that unsustainable patient-provider ratios, ideally 1:500–1,000, diminish CHV effectiveness and drive burnout.^36^ Similarly, Golob et al. (2022) linked understaffing in South Africa to 60% of unmet palliative care needs, while Ethiopia and India report parallel attrition trends due to overload.^37^

Uganda’s scale intensifies these challenges. With 179,175 CHVs serving 57,735 villages, this works out to about three CHVs per village.^14^ Around 30% remain untrained, with pronounced regional disparities. Burnout is exacerbated by minimal support for large populations, reflected in Northern Region dropout rates reaching 38%. Institutional weaknesses include unclear referral pathways, fragmented NGO trainings, and inconsistent supervision, with only 70% coverage and no standardisation.^14^ Potential responses include recalibrating population: CHV ratios toward evidence-based targets (e.g., 1:750),^36^ implementing task-shifting for administrative roles, and accelerating recruitment in underserved regions.^38^

The study also reveals a precarious financial reality where compensation is often inadequate, delayed, or absent. This instability undermines workforce sustainability and drives attrition. Current compensation systems fail to meet basic livelihood needs, turning volunteerism into an economic burden. Combined financial and emotional strain fuels burnout and reduces care quality. Existing models risk exploiting volunteer goodwill and obstructing the development of resilient health systems. A fundamental shift in incentive structures is therefore imperative toward predictable hybrid models integrating modest, reliable stipends with performance-linked non-cash rewards.^36,38,39^ Finally, coordination needs a national body to harmonise NGOs, secure funding for training and stipends, and pilot community recognition programmes to boost morale and sustainability.^40,41^

## Study strengths & limitations

This study possesses several notable strengths that enhance the validity and reliability of its findings. A key strength is the high ecological validity achieved by conducting interviews in the CHVs’ familiar environments, which created a safe and comfortable atmosphere for participants to speak openly, thereby reducing response bias. Furthermore, the research underwent rigorous quality assurance through multiple stages of critical feedback. This included four rounds of review prior to ethical approval, three formal assessments from university tutors and examiners, and additional peer feedback during academic sessions. Such extensive examination strengthened the study’s methodological rigour. Another significant strength was the use of in-depth, face-to-face interviews, which enabled the researchers to observe participants’ nonverbal cues, such as body language and facial expressions. This approach facilitated deeper probing during discussions, yielding richer qualitative data about the volunteers’ needs and experiences.

However, the study also has several limitations that must be acknowledged. Firstly, the researchers were unable to independently select participants due to the absence of a comprehensive list of CHVs, relying instead on a team leader to identify eligible volunteers. This introduced potential selection bias, compounded by the fact that fewer CHVs than anticipated met the language proficiency criteria, resulting in a smaller sample size. Furthermore, data saturation was not achieved, as new information and unmet needs continued to be identified from each interview. This limitation was primarily due to the restricted number of participants which prevented the inclusion of additional CHVs from other villages. With respect to transferability, the findings are context-specific, reflecting the lived experiences of CHVs within a single Ugandan parish. Nonetheless, they may be transferable to other low-resource sub-Saharan African settings with similar CHV programme structures, healthcare infrastructure challenges, and palliative care integration gaps. Factors that may limit transferability include local governance structures, NGO presence, and the degree of palliative care integration within national health systems. Contextual adaptation is therefore recommended. Another limitation of this study is that the interview guide was not formally pilot-tested or validated prior to data collection. Although we consulted content experts and a CHV to ensure the guide appeared valid on the surface, we did not conduct a formal pilot phase. We recognise that this might have affected the consistency of our data collection in the early stages of the study.

## Conclusion

This study identified significant needs of CHVs across four key domains essential for strengthening palliative care delivery in Uganda: Training and Education, Resource, Communication and Coordination, and Workforce Sustainability. CHVs function as a cornerstone of community-based palliative care, providing indispensable services including pain and symptom management, psychosocial support, health education, patient advocacy, and facilitating linkages to formal health services for patients with life-limiting illnesses. However, systemic challenges persist which limit their full potential. Addressing the identified gaps in training and resource allocation is necessary to overcome major barriers to equitable, high-quality palliative care access in this low-resource setting.

## Recommendations

Based on the findings, a multidimensional strategy is recommended to strengthen the capacity of CHVs in delivering palliative care in Uganda. Standardised, competency-based palliative care training, covering symptom management, psychosocial support, and chronic disease care, should be integrated into the national curriculum, supplemented by regular refresher courses and mentorship. Resource provision is vital, including “baseline kits” of essential and medical supplies, as well as monthly transport stipends to improve access. Digital tools such as mHealth applications can enhance communication, coordination, and data reporting. Workforce expansion to optimal CHV-to-household ratios and hybrid incentive models combining stipends and non-cash rewards are critical to sustain motivation and reduce attrition.

## Future research

Future research should prioritise patient-centered needs assessments in underserved areas, evaluating gaps in medication access and psychosocial support. Mixed-methods studies should assess how standardised interventions (e.g., training, equipment kits, and incentive models) impact CHV retention and care quality. Additionally, research should explore integrating palliative care into Uganda’s CHVs framework, testing mHealth tools for CHV support and investigating strategies to reduce burnout.

## Supplemental material

Supplemental material - Bridging the gap: Understanding the needs of community health volunteers to enhance palliative care delivery Supplemental material for Bridging the gap: Understanding the needs of community health volunteers to enhance palliative care delivery by Mark Donald Mwesiga, Lisa Christine Irumba, Christine Siew Lan Low, Christie van Veen, Nena van de Weijer, Suzanne Gros, Dorien van de Ven, Wout Linsen, Joyce Zalwango, Dianah Basirika, Rose Kiwanuka, John Muwonge, Mwazi Batuli and Gloria Kirungi Kasozi in Palliative Care and Social Practice.

## Data Availability

The interview data that support the findings of this study cannot be made publicly available due to the sensitive nature of this data, as per the ethics approval obtained.*

## References

[1] Olaniran A Smith H Unkels R , et al. Who is a community health worker? – a systematic review of definitions. Global Health Action 2017; 10(1): 1272223. 10.1080/16549716.2017.1272223 28222653 PMC5328349

[2] Laleman G Kegels G Marchal B , et al. The contribution of international health volunteers to the health workforce in sub-Saharan Africa. Human Resources for Health 2007; 5(1): 19. 10.1186/1478-4491-5-19 17672889 PMC1971668

[3] World Health Organization (WHO) . Declaration of Alma-Ata 1978. Available from: https://www.who.int/teams/social-determinants-of-health/declaration-of-alma-ata

[4] World Health Organization (WHO) . What do we know about community health workers? A systematic review of existing reviews 2021, Available from: https://www.who.int/publications/i/item/what-do-we-know-about-community-health-workers-a-systematic-review-of-existing-reviews

[5] South J Meah A Bagnall AM , et al. Dimensions of lay health worker programmes: results of a scoping study and production of a descriptive framework. Glob Health Promot 2013; 20(1): 5–15. 10.1177/1757975912464248 23563775

[6] O'Brien MJ Squires AP Bixby RA , et al. Role development of community health workers: an examination of selection and training processes in the intervention literature. Am J Prev Med 2009; 37(6 Suppl 1): S262–S269. 10.1016/j.amepre.2009.08.011 19896028 PMC2856599

[7] Bhutta ZA Lassi ZS Pariyo G , et al. Global experience of community health workers for delivery of health related millennium development goals: a systematic review, country case studies, and recommendations for integration into national health systems. Global health workforce Alliance 2010; 1(249): 61.

[8] UmoH U . Health Sector Strategic and Investment Plan: Promoting People’s Health to Enhance Socio-economic Development 2010/11–2014/15. Uganda Ministry of Health(UMoH) and UNICEF, 2010. https://www.unicef_org/uganda/HSSIP_Final_pdf

[9] Musinguzi LK Turinawe EB Rwemisisi JT , et al. Linking communities to formal health care providers through village health teams in rural Uganda: lessons from linking social capital. Human Resources for Health 2017; 15(1): 4. 10.1186/s12960-016-0177-9 28077148 PMC5225547

[10] Musoke D Ndejjo R Atusingwize E , et al. Performance of community health workers and associated factors in a rural community in Wakiso district, Uganda. Afr Health Sci 2019; 19(3): 2784–2797. 10.4314/ahs.v19i3.55 32127852 PMC7040253

[11] Ministry of Health (MOH) . Annual health sector performance report, financial year 2019/20. Ministry of Health Kampala, 2020.

[12] Kimbugwe G Mshilla M Oluka D , et al. Challenges Faced by Village Health Teams (VHTs) in Amuru, Gulu and Pader Districts in Northern Uganda. Open J Prev Med 2014; 4(9): 740–750. 10.4236/ojpm.2014.49084 26301128 PMC4542049

[13] Lusambili AM Nyanja N Chabeda SV , et al. Community health volunteers challenges and preferred income generating activities for sustainability: a qualitative case study of rural Kilifi, Kenya. BMC Health Services Research 2021; 21(1): 642. 10.1186/s12913-021-06693-w 34217281 PMC8254366

[14] Ministry of Health (MOH) Uganda . National Village Health Teams(VHT) Assessment in Uganda. Kampala, 2015.

[15] Babughirana G Muhirwe Barungi L Kimurahebwe C . Village health team functionality in Uganda: Implications for community system effectiveness. Science Journal of Public Health, 2016; 4(2): 117–126. 10.11648/j.sjph.20160402.16

[16] Ministry of Health (MOH) . National Village Health Teams(VHT) Assessment in Uganda Kampala. Uganda Ministry of Health (MOH), 2015. Available from: https://library.health.go.ug/index.php/community-health/health-education/national-village-health-teamsvht-assessment-uganda

[17] Ministry of Health (MOH) . Situation analysis village health teams Kampala. Ministry of Health, 2009. Available from: https://library.health.go.ug/sites/default/files/resources/Situation_Analysis_Village_Health_Teams_Uganda_2009.pdf

[18] Reid E Lukoma M Ho D , et al. Palliative care needs and barriers in an urban Ugandan Emergency Department: A mixed-methods survey of emergency healthcare workers and patients. Afr J Emerg Med 2023; 13(4): 339–344. 10.1016/j.afjem.2023.11.005 38162896 PMC10757186

[19] Hennink MM Kaiser BN Marconi VC . Code saturation versus meaning saturation: how many interviews are enough? Qualitative health research 2017; 27(4): 591–608. 10.1177/1049732316665344 27670770 PMC9359070

[20] Richards L Morse JM . Readme first for a user's guide to qualitative methods. 3rd ed. 2013 Los Angeles, CA: SAGE.

[21] Bingham AJ Witkowsky P . Deductive and inductive approaches to qualitative data analysis. Analyzing and interpreting qualitative data: After the interview. 2021;1:133–146.

[22] Bingham AJ . From Data Management to Actionable Findings: A Five-Phase Process of Qualitative Data Analysis. International Journal of Qualitative Methods 2023; 22: 16094069231183620. 10.1177/16094069231183620

[23] Musoke D Ssemugabo C Ndejjo R , et al. Strengthening the community health worker programme for health improvement through enhancing training, supervision and motivation in Wakiso district, Uganda. BMC Research Notes 2019; 12(1): 812. 10.1186/s13104-019-4851-6 31852520 PMC6921412

[24] Lukyamuzi Z Ssuna B Mirembe RN , et al. Experiences and challenges of using community health worker-led mechanism in supporting HIV disclosure among adults living with HIV in heterosexual relationships in the rural Uganda. AIDS Research and Therapy 2023; 20(1): 14. 10.1186/s12981-023-00508-0 36906557 PMC10008611

[25] Kroeze VA Pithan A Kupershmidt S , et al. Integration of Community Health Workers into Palliative Care Teams: A Scoping Review. West J Nurs Res 2025; 47(2): 110–124. 10.1177/01939459241304520 39707797

[26] Ministry of Health (MOH) Uganda . COMMUNITY HEALTH EXTENSION WORKERS (CHEWs). Kampala, 2018. Available from: https://library.health.go.ug/file-download/download/public/1269

[27] Källander K Tibenderana JK Akpogheneta OJ , et al. Mobile health (mHealth) approaches and lessons for increased performance and retention of community health workers in low- and middle-income countries: a review. J Med Internet Res 2013; 15(1): e17. 10.2196/jmir.2130 23353680 PMC3636306

[28] Agarwal S Abuya T Kintu R , et al. Understanding community health worker incentive preferences in Uganda using a discrete choice experiment. J Glob Health 2021; 11: 07005. 10.7189/jogh.11.07005 33763219 PMC7956012

[29] Mays DC O'Neil EJ Mworozi EA , et al. Supporting and retaining Village Health Teams: an assessment of a community health worker program in two Ugandan districts. Int J Equity Health 2017; 16(1): 129. 10.1186/s12939-017-0619-6 28728553 PMC5520299

[30] Mwaniki AW Nyaboga JM Mecha EO , et al. Performance of community health volunteers during the COVID-19 pandemic: assessing the enablers and challenges in Machakos County, Kenya. Prim Health Care Res Dev 2025; 26: e65. 10.1017/S1463423625100248 40734622 PMC12455361

[31] Kolla A Lim S Zanowiak J , et al. The Role of Health Informatics in Facilitating Communication Strategies for Community Health Workers in Clinical Settings: A Scoping Review. J Public Health Manag Pract 2021; 27(3): e107. 10.1097/PHH.0000000000001092 33512874 PMC7994181

[32] Babughirana G Muhirwe Barungi L Kimurahebwe C . Village Health Team Functionality in Uganda: Implications for Community System Effectiveness. Science Journal of Public Health 2016; 4(2): 117–126.

[33] Tilahun D Fekadu A Tekola B , et al. Ethiopian community health workers’ beliefs and attitudes towards children with autism: Impact of a brief training intervention. Autism 2019; 23(1): 39–49. 10.1177/1362361317730298 28945112

[34] Uganda Bureau of Statistics (UBOS) . Uganda National Household Survey 2019/2020. Kampala, Uganda: UBOS, 2021. Available from: https://www.ubos.org/wp-content/uploads/publications/06_2021UNHS2019-20_presentation.pdf

[35] Labrique A Vasudevan L Mehl G , et al. Digital Health and Health Systems of the Future. Glob Health Sci Pract 2018; 6(Suppl 1): S1–s4. 10.9745/GHSP-D-18-00342 30305334 PMC6203414

[36] Jaskiewicz W Tulenko K . Increasing community health worker productivity and effectiveness: a review of the influence of the work environment. Human Resources for Health 2012; 10(1): 38. 10.1186/1478-4491-10-38 23017131 PMC3472248

[37] Golob S Zilinyi R Godfrey S , et al. The prevalence of palliative care consultation in deceased COVID-19 patients and its association with end-of-life care. Journal of Palliative Medicine 2022; 25(1): 70–74. 10.1089/jpm.2021.0049 34191603

[38] World Health Organisation (WHO) . Task shifting: Rational redistribution of tasks among health workforce teams: Global recommendations and guidelines. WHO, 2008. Available from: https://iris.who.int/bitstream/handle/10665/341139/9789240024229-eng.pdf

[39] World Health Organisation (WHO) . What do we know about community health workers? A systematic review of existing reviews. WHO, 2020. Available from: https://iris.who.int/bitstream/handle/10665/340717/9789241512022-eng.pdf

[40] Schneider H . The governance of national community health worker programmes in low-and middle-income countries: an empirically based framework of governance principles, purposes and tasks. International Journal of Health Policy and Management 2018; 8(1): 18–27. 10.15171/ijhpm.2018.92 PMC6358641 30709099

[41] Cometto G Ford N Pfaffman-Zambruni J , et al. Health policy and system support to optimise community health worker programmes: an abridged WHO guideline. The Lancet Global Health 2018; 6(12): e1397–e1404. 10.1016/S2214-109X(18)30482-0 30430994

