# Supplementary material for: Bridging the gap: Understanding the needs of community health volunteers to enhance palliative care delivery
Source: Palliat Care Soc Pract. 2026 Aug 23;20:26323524261473935. doi: 10.1177/26323524261473935 (PMC13504121; doi:10.1177/26323524261473935)
Supplement: Supplementary file 1 [file sj-pdf-1-pcr-10.1177_26323524261473935.pdf]

## *Appendix 1 - Ethical Approval*

# Hospice Africa Uganda Research Ethics Committee


**HOSPICE AFRICA UGANDA**
P.O. Box 7757, Kampala - Uganda
Tel: +256 392 766867 and +256 701 968098
+256 392 174892
Email: infoug@hospice-africa.org

**LITTLE HOSPICE HOIMA**
P.O. Box 430, Hoima - Uganda
Tel: +256 700 457071 and +256 772 090218

**MOBILE HOSPICE MBARARA**
P.O. Box 1030, Mbarara - Uganda
Tel: +256 772 384038 and +256 705 169125

**HAU/IHPCA**

Email: research@hospice-africa.org Website: www.hospice-africa.org/uganda
Facebook: Hospice Africa Uganda Twitter: @HospiceAfricaUg


HOSPICE AFRICA UGANDA
APPROVED & VALID UNTIL
09 NOV 2024
RESEARCH & ETHICS COMMITTEE
P.O. BOX 7757, KAMPALA (U)

9th / 11/2023

To: Christie Vanveen

HAN University of Applied Sciences

Type: Initial Review

**Re: HAU-2023-02A, NEEDS OF COMMUNITY HEALTH VOLUNTEERS TO IMPROVE THE PALLIATIVE CARE THEY ARE GIVING TO THEIR PATIENTS IN THE MUTUNGO PARISH, WAKISO DISTRICT, UGANDA**

I am pleased to inform you that the Hospice Africa Uganda REC approved the above-referenced study through Fast Track review.
The approval is from **9th /11 /2023** to **9th 11/2024.**

As Principal Investigator of the research, you are responsible for fulfilling the following requirements.

1. All co-investigators must be informed of the research status.
2. Changes, amendments, and addenda to the protocol or the consent form must be submitted to the REC for review and approval **before activating** the changes.
3. Reports of unanticipated problems involving risks to participants or any new information that could change the risk-benefit: ratio must be submitted to the REC.
4. Only approved consent forms should be used in participant enrollments. All consent forms signed by participants and/or witnesses should be retained on file. The REC may conduct audits of all study records, and consent documentation may be part of such audits.
5. Continuing review application must be submitted to the REC **eight weeks** before the expiration date of **9th /11/2024** to continue the study beyond the approved period. Failure to submit a continuing review application in a timely fashion may result in suspension or termination of the study.
6. The REC application number assigned to the research should be cited in any correspondence with the REC of record.

The following is the list of all documents approved in this application by Hospice Africa Uganda REC:

**HOSPICE AFRICA UGANDA**
P.O. Box 7757, Kampala - Uganda
Tel: +256 392 766667 and +256 701 968098
+256 392 174892
Email: infoug@hospice-africa.org

**LITTLE HOSPICE HOIMA**
P.O. Box 430, Hoima - Uganda
Tel: +256 700 457971 and +256 772 800218

**MOBILE HOSPICE MBARARA**
P.O. Box 1038, Mbarara - Uganda
Tel:+256 772 304038 and +256 705 101125

HAU/IHPCA

Email: research@hospice-africa.org Website: www.hospice-africa.org/uganda
Facebook: Hospice Africa Uganda Twitter: @HospiceAfricaUg


| No | Document Title | Language | Version Number | Version Date |
|---|---|---|---|---|
| 1. | Protocol | English | 1 | 2023-11- 08 |
| 2. | Data Collection Tool | English | 1 | 2023- 11-08 |
| 3. | Consent form | English | 1 | 2023- 11- 08 |

Yours sincerely,

[signature]

Prof Wilson Acuda
Chairman HAUREC
wsacuda@hospiceafrica.or.ug
0703184199

HOSPICE AFRICA UGANDA
APPROVED & VALID UNTIL
09 NOV 2024
RESEARCH & ETHICS COMMITTEE
P.O. BOX 7757, KAMPALA (U)



Appendix 2- Interview guide

**Introduction:**

Good morning/afternoon/evening, it is lovely to meet you.

First I want to thank you for speaking to us. We really appreciate it!

We are Suzanne, Christie and Nena and we are three nursing students of the University of Applied Sciences in Nijmegen, the Netherlands. We are here to conduct an interview with you about your work as a community health volunteer. We are excited to hear about it.

The Palliative Care Association of Uganda signalled some challenges in giving palliative care. That is why they asked us to do research about it. This led to the question: What do community health volunteers need, to improve the palliative care they are giving to their patients? We are researching what these needs are.

Our research and this interview is completely anonymous. During the interview we will record this interview. Are you okay with all of this?

Thank you so much again for having us. Let's start the interview here.

| **Aim of the interview:**<br>Gain understanding about what community health volunteers need to improve the palliative care they are giving to their patients. | | |
|---|---|---|
| **Method of documentation:**<br>● Audio recording<br>● Written notes | | |
| **Topic** | **Question example** | **Question type** |
| Introductory | What is your age? | Introductory question |
| Introductory | How long have you been a CHV? | Introductory question |
| Introductory | How many days a week do you work as a CHV? | Introductory question |
| Introductory | How long is a working day? | Introductory question |
| Introductory | How many times are you in contact with palliative care during your work as a CHV? | Introductory |

| | | |
|---|---|---|
| | | question |
| Introductory | What are you doing while giving palliative care? | Introductory question |
| Introductory | What kind of education did you have when you were younger? | Introductory question |
| Research question | What do you need, to improve the palliative care you are giving? | |
| Education | Why did you become a CHV? | Direct question |
| Education | What did you have to do to become a CHV? | Direct question |
| Education | Did you receive any training before you became a CHV? | Closed question |
| Education | If yes, what did this training look like? | Follow-up question |
| Education | If yes, what kind of training have you received in palliative care? | Follow-up question |
| Resources | How are you going to see a patient? | Direct question |
| Resources | What do you bring when you are visiting a patient who needs palliative care? | Direct question |
| Resources | Do you document somewhere what care you have given to a patient? | Closed question |
| Resources | If yes, what does this documentation look like? | Follow-up question |
| Resources | What do you receive for being a CHV? | Direct question |

| | | |
|---|---|---|
| Resources | What kind of compensation do you receive for your services from the patient? | Direct question |
| Communication and coordination | Do you collaborate with other people? | Closed question |
| Communication and coordination | What does this collaboration look like? | Follow-up question |
| Communication and coordination | How do you communicate with other healthcare providers and agencies involved in patient care? | Direct question |
| Communication and coordination | How do people reach you? | Direct question |
| Individual factors | Do you think the work you are doing as a CHV is intense? | Opinion question |
| Individual factors | Can you explain why (not)? | Follow-up question |
| All topics | Am I interpreting correctly that you mean X? | Interpretation question |
| All topics | I would like to talk a little more about X, is that okay with you? | Structuring question |
| All topics | Did you see any changes in the work as a CHV during the time you have worked? | Direct question |

Analysis of Theme 1: Education

| No | Interview | Text | Codes | Subtheme | The |
|---|---|---|---|---|---|
| 482 | R5 | Even us as volunteers we want to do studies. we take us also and see those who are coming from other country and are also of suffering with the same illness and then share information. we share with them we volunteer and chose one, two, three or five and take them to your country and see how they are also taking care of their life and then we come back and improve it here | Wanting to study<br>Working with other countries | Needs for education | |

| | | | | | |
|---|---|---|---|---|---|
| 22 | R1 | Me as a volunteer, currently as a volunteer I need to know. round for me to learn more. I finished my bachelors in 2021 and finished the bachelor of informative science technology and management. then I picked up to go do a certificate for public health. So, I wish to learn more. When we are here there are things that (11.44) we don't get that .... (11.47) We learn they need still go to help us. And that run this works effectively. We have the challenge of which we can't ...... Meaning we think more in other opportunities that are oblivious that platform. Require also for us to study more, to come and help our people. You have the passion to get the challenge of ... | Early training<br><br>Learning more & opportunities | Amount of training<br><br>Motivation | |
| 30 | R1 | So, it not that effective and lacking, still lacking. | Lack of training | Amount of training | |
| 62 | R1 | Actually, it makes us also a way of something that not has yet been done and put into our program. When your research people like you coming on the board. When we interact and share ideas, we find that we get to know the fine things, and we learn something. Regardless of that. if people are there, they will also extend services. People are willing but they don't have, they need some little adjustment in their training for such services. To come up and to say such organisation who can give a special training to come around and find that. What I've learnt, if I get the opportunity, it will motivate someone who is behind and come and say let me get of that. Such for a widen our number and will be able to fit in the community we serve them to our expectation but that is the reality I wish to go further education regardless and help safely and when we go out it is making our people to have a good life and be able to give back 41.00... our expectation, if we make them this, why do we also extend to another. How can we make such a person come and learn different skills come and do, and then this person is not able to,<br><br>How can we make that person come and learn some different skills. …They will learn is something. Make just a little better. To connect with such person, what learn something. Come and teach them.<br><br>When you have, you will see the community improving. So have a different idea we bring to our | Training of CHV<br><br>Learning various skills<br><br>Increase knowledge & creative new ideas<br><br>Motivation<br><br>Reciprocal effect<br><br>Further education | Type of training<br><br>Innovation<br><br>Motivation | |

| | | | | | |
|---|---|---|---|---|---|
| 275 | | people… Let them learn something in that the next day for them improves and they feel safe and can afford the medicine. The fact that we have a lot of challenges for this services and other people have also challenges but we can't meet the expectations or their challenges of a 100%.<br><br>So, it would be better when we learn other skills (referring to life functioning skills), so we come and equip them, and they be able to run their life. | | | |
| 114 | R2 | there were some trainings which we did by our boss and when I used to be at that side is also used to have some trainings. | Early training | Amount of training | |
| 116 | R2 | These trainings were there but now we are not having because of funds. We used to have trainings but now we do not have. We are not having I think it is because of money that we are not getting training. | No funds for training<br><br>Lack of money | Training Resource | |
| 120 | R2 | When during Covid we train how to handle this situation of Covid. How we need to protect ourselves and how to report the cases when they arrise in the community and where to report the cases. | Content of training | Type of training | |
| 275 | R3 | Yeahhh, I get it the training in palliative care. I go there, I get the training, hmm, and then the time when we start the work to be a local counsellor, you get the training. All of us get the training. With other districts, they tell us how to be with those | Training of CHV | Type of training | |

| | | | | | |
|---|---|---|---|---|---|
| | | people. That is, it. | | | |
| 279 | R3 | I get it, when I get it, it was like one week. Cause I used to go there but even the transport is a problem between, but I get something. Yeah | Length of training | Amount of training | |
| 308 | R4 | I just to train other community members. | Training other VHT<br><br>Motivate | Training | |
| 320 | R4 | So it would be better when we learn other skills, so we come and equip them, and they be able to run their life. | Training palliative care<br><br>Training needs | Type of training | |
| 322 | R4 | yeah yeah, yeah how to counsel them and how to yeah that they take their medicine you know and remind them go to the hospital | Training palliative care | Type of training | |
| 326 | R4 | No, one day a month training | Length of training | Amount of training | |
| 329 | R4 | No, they stop | Stop training | Amount of training | |
| 374 | R4 | I got training on how to consult and recognise people (with palliative care needs) in the community…We are missing the skills (referring to palliative care knowledge & skills) because some of us (referring to the people in the community) are not in good condition. I think we could get some skills that help us, so that when we have skills, we can teach others because volunteers want to help others because it is in our blood. | Missing skills<br><br>Needed skills<br><br>Motivation to share knowledge | Types of training<br><br>Motivation | |
| 378 | R4 | yeah hhm hhm, because when you have when we get a project, we look for other who can teach us | Teaching others<br><br>Motivation | Training<br><br>Motivation | |

| | | | | | |
|---|---|---|---|---|---|
| 416 | R5 | uhm on the palliative care, I got counselling. | counselling palliative care | Type of training | |
| 420 | R5 | The training I did it took like a week, a whole week but many times, we have training in here. they can call us, and we go back for the training and the training just to refresh us…the refreshment course is like four times a year. They can call us, and we go back for the training and the training. Just to refresh us as volunteers | Length of training<br><br>Refreshment course | Amount of training<br><br>Type of training | |
| 422 | R5 | the refreshment is like four times a year and that is for those ones who are having aids | Length of continuous learning | Amount of training | |
| 484 | R5 | Yes yes that will also help hhm you may choose some volunteers and then take them and see how taking care of patients then come back and tell them that idea an tell this and this | Learn from others<br><br>New ideas | Type of training | |
| 525 | R6 | Yes we trained for 2 years. On those days we had a chance to bring nurses from Mulago Hospital. | Length of training | Amount of training | |
| 531 | R6 | Yes. And we get some seminars for three times a quarter to add information. | Follow-up training | Amount of training | |
| 535 | R6 | The training. The training look like we want. We have 6 villages. We praise our friends who want in PC, they come and get teached some information. | Kind of training | Type of training | |
| 641 | R7 | To be more educated because some patient may come as HIV patient and they want something, they want more people to understand them because someone can come when stressed, you forget that kind of diseases, you can feel if you are going to die at that moment. I need more education in that. | More education needed<br><br>Refresher topic on diseases | Type of training<br><br>Gaps in training | |
| 643 | R7 | No, nothing because they had just chosen me because I was working in the hospital. | No training before | Amount of training | |
| 774 | R8 | they thought us how to things under five years; to give medicine to children. and we give water and when a kid had malaria or pneumonia, we know how to respond to, and we also refer when mother come to us for help we help them to go to other health centre. | Teaching contents | Type of training | |

| | | | | | |
|---|---|---|---|---|---|
| 776 | R8 | yeah we sometimes they call us for refresher training, and they also train how to conduct a malaria test, how to take sample of malaria of sick children | Refresh training<br><br>Paediatric | Amount of training<br><br>Types of training | |
| 806 | R8 | Yeah, we want to get more training (referring to palliative care knowledge & skills), beyond what we have | Increase Knowledge & improve skills | Amount of training | |
| 808 | R8 | …we need to reach that level where the minister knows how much work and the woman with HIV has and those who need to know to get more than only the five years. | Lack of training<br><br>Length of training | Amount of training | |
| 809 | R3 | so, you get only training from 0 till 5. and more about the older people. | Length of training | Length of training | |
| 814 | R8 | yeah, training and motivation | Training<br><br>Motivation | Training<br><br>Motivation | |
| 832 | R8 | …i think it would be an opportunity to maybe i think to choose those VHT who are great and take them somewhere like Netherlands for upgrading and they learn more about medicine and they have now no to upgrade themselves. you are taking a number of VHTs to go and upgrade in another country | Learning from other countries<br><br>Higher education | Type of training<br><br>Motivation | |
| 833 | R3 | So, like for example you like twenty people go to the Netherlands and then come back and share | VHT go to other country | Training | |
| 768 | R8 | yes yes i got a training of unity it was for three weeks as a volunteer worker | Length of training | Length of training | |

| | | | | | |
|---|---|---|---|---|---|
| 28 | R1 | Yes they do provide us that. Like it is education on devision level. They always call us we as volunteers, the health carers of within our areas. They will call us; they can train us. How to handle data for such patients.<br><br>Sometimes they have not .... yet to anyone to learn. So how to manage such data, they always give us such data and how to make it safe and how can we help such a person before ... at least they give us that, but which is not effective that much because they sometimes only give us a day training program. Of which, I myself, seem to have gone beyond that. And how often do they do it. You find sometimes are only once in a month or half a year or maybe only twice, which is not effective to this service. How I wish to go beyond that and to get the best men. When you have the materials, 14.04 this one we are doing it just for ... Something on the board to learn as a class. We need to have the materials. First aid kits, do they provide us? Those are challenges. We are lacking ... | Early training<br><br>Lack of material<br><br>Type of training<br><br>Length of training<br><br>Training effectiveness | Amount of training<br><br>Medical Item<br><br>Resources<br><br>Type of training | |
| 376 | R4 | Yeah, because when we have skills, we do not have to be begging, begging, for money but when there is a skill a project that is where we can get some money | Missing skills<br><br>Finance | Type of training<br><br>Cost | |

Manuscript Title: Bridging the Gap: Understanding the Needs of Community Health Volunteers to Enhance Palliative Care Delivery

Consolidated criteria for reporting qualitative studies (COREQ): 32-item checklist

Developed from:
Tong A, Sainsbury P, Craig J. Consolidated criteria for reporting qualitative research (COREQ): a 32-item checklist for interviews and focus groups. *International Journal for Quality in Health Care*. 2007. Volume 19, Number 6: pp. 349 – 357

| No. Item | Guide questions/description | Reported on Page # |
|---|---|---|
| Domain 1: Research team and reflexivity | | |
| *Personal Characteristics* | | |
| 1. Inter viewer/facilitator | Which author/s conducted the interview or | Three interviewers |

| | | |
|---|---|---|
| | focus group? | conducted the interview with the patients:<br>Christie van Veen<br>Nena van de Weijer<br>Suzanne Gros |
| 2. Credentials | What were the researcher's credentials? E.g. PhD, MD | Bachelors |
| 3. Occupation | What was their occupation at the time of the study? | Medical Students |
| 4. Gender | Was the researcher male or female? | Female |
| 5. Experience and training | What experience or training did the researcher have? | The researchers were taught in their medical training about conducting interviews and were guided by their experienced university supervisors and a CHV. On top of that, they were also guided by Hospice Africa Uganda Research & Ethics Committee (HAUREC), Commissioner of Palliative Care at the Ministry of Health in Uganda and an advisor of Palliative Care Association of Uganda (PCAU) to gain more insight of the healthcare process and research training in Uganda. |
| *Relationship with participants* | | |
| 6. Relationship established | Was a relationship established prior to study commencement? | No |

| | | |
|---|---|---|
| 7. Participant knowledge of the interviewer | What did the participants know about the researcher? e.g. personal goals, reasons for doing the research | Participants knew nothing about the researchers |
| 8. Interviewer characteristics | What characteristics were reported about the interviewer/facilitator? e.g. Bias, assumptions, reasons and interests in the research topic | There was no bias or assumptions in the research |

| | | |
|---|---|---|
| Domain 2: study design | | |
| *Theoretical framework* | | |
| 9. Methodological orientation and Theory | What methodological orientation was stated to underpin the study? e.g. grounded theory, discourse analysis, ethnography, phenomenology, content analysis | Included in Method section: Data analysis<br><br>(Please refer to page No.8) |
| *Participant selection* | | |
| 10. Sampling | How were participants selected? e.g. purposive, convenience, consecutive, snowball | Included in Method section: i. Selection and Description of Participants<br><br>(Please refer to page No.7) |
| 11. Method of approach | How were participants approached? e.g. face-to-face, telephone, mail, email | Included in Method section: i. Selection and Description of Participants & ii. Data Collection.<br><br>(Please refer to page No.7) |
| 12. Sample size | How many participants were in the study? | Included in Results section: "Social demographics of respondents"<br><br>(Please refer to page No.9) |
| 13. Non-participation | How many people refused to participate or dropped out? Reasons? | No participants refuse to participate or drop out. |
| *Setting* | | |
| 14. Setting of data collection | Where was the data collected? e.g. home, clinic, workplace | Included in Method section: Data collection<br><br>(Please refer to page No.7 & 8) |
| 15. Presence of non-participants | Was anyone else present besides the participants and researchers? | No other individuals were present in interviews |
| 16. Description of sample | What are the important characteristics of the sample? e.g. demographic data, date | Included in Method section: Data collection<br><br>(Please refer to page No.7) |

| | | |
|---|---|---|
| *Data collection* | | |
| 17. Interview guide | Were questions, prompts, guides provided by the authors? Was it pilot tested? | Included in Method section: Data collection<br><br>(Please refer to page No.7 & Appendix 2) |
| 18. Repeat interviews | Were repeat inter views carried out? If yes, how many? | Repeating interviews were not carried out in this study and have therefore not been reported. |
| 19. Audio/visual recording | Did the research use audio or visual recording to collect the data? | Included in Methods section: Data collection<br><br>(Please refer to page No.7) |
| 20. Field notes | Were field notes made during and/or after the inter view or focus group? | Included in Methods section: Data collection<br><br>(Please refer to page No.7-8) |
| 21. Duration | What was the duration of the inter views or focus group? | Included in Methods section: Data collection<br><br>(Please refer to page No.7-8) |
| 22. Data saturation | Was data saturation discussed? | Included in Study Strengths & Limitations section.<br><br>(Please refer to page No.21) |
| 23. Transcripts returned | Were transcripts returned to participants for comment and/or correction? | Included in Methods section: Data collection<br><br>(Please refer to page No.7-8) |
| Domain 3: analysis and findings | | |

| | | |
|---|---|---|
| *Data analysis* | | |
| 24. Number of data coders | How many data coders coded the data? | Included in Methods section: Data Analysis<br><br>(Please refer to page No.8) |
| 25. Description of the coding tree | Did authors provide a description of the coding tree? | Included in Methods section: Data Analysis<br><br>(Please refer to page No.8 and Appendix 3) |
| 26. Derivation of themes | Were themes identified in advance or derived from the data? | Included in Methods section: Data Analysis<br><br>(Please refer to page No.8) |
| 27. Software | What software, if applicable, was used to manage the data? | Included in Methods section: Data Analysis<br><br>(Please refer to page No.8) |
| 28. Participant checking | Did participants provide feedback on the findings? | Included in Methods section: Data collection<br><br>(Please refer to page No.8) |
| *Reporting* | | |
| 29. Quotations presented | Were participant quotations presented to illustrate the themes/findings? Was each quotation identified? e.g. participant number | Yes, demonstrated throughout the Result section.<br><br>(Please refer to page No. 9-18) |
| 30. Data and findings consistent | Was there consistency between the data presented and the findings? | Yes, demonstrated throughout the Result & Discussion section.<br><br>(Please refer to page No. 9-22) |

| | | |
|---|---|---|
| 31. Clarity of major themes | Were major themes clearly presented in the findings? | Yes, demonstrated throughout findings. Themes rather than descriptive details have been included.<br><br>(Please refer to page No. 9-18) |
| 32. Clarity of minor themes | Is there a description of diverse cases or discussion of minor themes? | No, this was not included. |
